# Expression of the functional recombinant human glycosyltransferase GalNAcT2 in *Escherichia coli*

**DOI:** 10.1186/s12934-014-0186-0

**Published:** 2015-01-13

**Authors:** Jennifer Lauber, René Handrick, Sebastian Leptihn, Peter Dürre, Sabine Gaisser

**Affiliations:** Institute of Applied Biotechnology, Biberach University of Applied Sciences, Biberach, Germany; Institute of Microbiology and Molecular Biology, University of Hohenheim, Stuttgart, Germany; Institute of Microbiology and Biotechnology, University of Ulm, Ulm, Germany

**Keywords:** Protein expression, *Escherichia coli*, Glycosyltransferase GalNAcT2, Erv1p/PDI co-expression, *in vitro* glycosylation, Filgrastim, Recombinant glycosyltransferase, Enzymatic activity glycosyltransferase, Secondary structure glycosyltransferase

## Abstract

**Background:**

Recombinant protein-based therapeutics have become indispensable for the treatment of many diseases. They are produced using well-established expression systems based on bacteria, yeast, insect and mammalian cells. The majority of therapeutic proteins are glycoproteins and therefore the post-translational attachment of sugar residues is required. The development of an engineered *Escherichia coli*-based expression system for production of human glycoproteins could potentially lead to increased yields, as well as significant decreases in processing time and costs.

**Results:**

This work describes the expression of functional human-derived glycosyltransferase UDP-GalNAc:polypeptide N-acetylgalactosaminyltransferase 2 (GalNAcT2) in a recombinant *E. coli* strain.

For expression, a codon-optimised gene encoding amino acids 52–571 of GalNAcT2 lacking the transmembrane N-terminal domain was inserted into a pET-23 derived vector encoding a polyhistidine-tag which was translationally fused to the N-terminus of the glycosyltransferase (HisDapGalNAcT2). The glycosyltransferase was produced in *E. coli* using a recently published expression system. Soluble HisDapGalNAcT2 produced in SHuffle® T7 host cells was purified using nickel affinity chromatography and was subsequently analysed by size exclusion chromatography coupled to multi-angle light scattering (SEC-MALS) and circular dichroism spectroscopy to determine molecular mass, folding state and thermal transitions of the protein. The activity of purified HisDapGalNAcT2 was monitored using a colorimetric assay based on the release of phosphate during transfer of glycosyl residues to a model acceptor peptide or, alternatively, to the granulocyte-colony stimulating growth factor (G-CSF). Modifications were assessed by Matrix Assisted Laser Desorption Ionization Time-of-flight Mass Spectrometry analysis (MALDI-TOF-MS) and Electrospray Mass Spectrometry analysis (ESI-MS). The results clearly indicate the glycosylation of the acceptor peptide and of G-CSF.

**Conclusion:**

In the present work, we isolated a human-derived glycosyltransferase by expressing soluble HisDapGalNAcT2 in *E. coli*. The functional activity of the enzyme was shown *in vitro*. Further investigations are needed to assess the potential of *in vivo* glycosylation in *E. coli*.

## Background

Recombinant protein therapeutics comprise a significant part of approved biotechnology-based medicines. Production systems for recombinant proteins include bacteria, yeast, insect and mammalian cells. Bacterial expression systems for recombinant human-derived proteins are widely used, but limited as most bacteria lack certain post-translational modification (PTM) mechanisms, including those for glycosylation [1]. In general, glycoproteins are produced in eukaryotic cell lines such as Chinese Hamster Ovary (CHO), murine myeloma (NS0) or Baby Hamster Kidney (BHK) [2]. Proteins stabilised by multiple disulfide bonds are preferentially expressed in eukaryotic CHO, yeast and insect cells, a production process that is often time consuming and cost-intensive [3].

About a third of the currently approved recombinant protein therapeutics are produced in *E. coli* strains [4]. The use of bacteria as expression hosts provides many advantages such as rapid growth, low-cost media, a versatility of cloning tools combined with the potential to produce compounds with high yield and quality [4,5]. So far, *E. coli*-based systems have been employed to express non-glycosylated proteins, however, the development of engineered strains allowing the expression of complex therapeutics including correct folding, disulfide bond formation, and glycosylation modifications are highly desirable and may potentially lead to a significant decrease in process time and costs [2,6].

Glycosylation represents one of the most common post-translational modifications, with N- and O-linked glycosylation requiring the activity of specific glycosyltransferases [2,7]. N-glycosylations take place on an amide nitrogen of the amino acid asparagine and O-glycosylations on mucin are initiated by the addition of the monosaccharide N-acetylgalactosamine to the hydroxyl group of the amino acids serine or threonine [8-11]. The presence, composition and pattern of glycosylation potentially influence various parameters such as glycoprotein functional activity, folding, stability and immunogenicity [6,12]. Various strategies have been pursued to isolate glycoengineered *E. coli* strains including the functional transfer of glycosylation pathways [6]. As an example, the production of eukaryotic N-glycoproteins has been demonstrated in *E. coli* engineered to express the N-glycosylation machinery of *Campylobacter jejuni* [11,13]. Similarly, the construction of an engineered *E. coli* strain has been described to successfully transfer glycans to target proteins by the expression of several heterologous glycosyltransferases from *Saccharomyces cerevisiae* in combination with the bacterial oligosaccharyltransferase PglB from *C. jejuni* [13]. The presence of O-linked glycosylation reactions in various bacterial strains such as *Neisseria* and *Acinetobacter* [14,15] further emphasises the future potential of glycoengineered bacteria as cell factories in industrial production processes [6]. Recently, a human sialyltransferase has been expressed successfully in optimised *E. coli* strains carrying mutations that provide an increased oxidative cytoplasmic environment or that co-express molecular chaperones [1]. The enzyme sialylates O-linked glycoproteins and the activity of the sialyltransferase catalysing the transfer of sialic acid onto an O-glycoprotein substrate has been demonstrated in a high throughput assay [1]. More recently, it has been shown that the pre-expression of the redox folding helper proteins sulfhydryl oxidase Erv1p derived from *S. cerevisiae* and the mature form of human protein disulfide isomerase PDI improves the production of soluble recombinant proteins with multiple disulfide bonds [16]. Erv1p represents a sulfhydryl oxidase and FAD-dependent catalyst of disulfide bonds present in the inter-membrane space of mitochondria [16]. Increased production of active proteins has been demonstrated in *E. coli* strains expressing Erv1p in the cytoplasm [16]. PDI is an ER-located protein in eukaryotes with multiple functions including disulfide bond formation, breakage and rearrangement [3,17].

The human-derived glycosyltransferase UDP-GalNAc:polypeptide N-acetylgalactosaminyltransferase 2 (GalNAcT2) catalyses the first step in mucin biosynthesis by transferring N-acetylgalactosamine (GalNAc) from the sugar donor uridine-5′-diphospho-N-acetylgalactosamine (UDP-GalNAc) to serine and threonine residues [18,19]. GalNAcT2 is a typical type II transmembrane protein anchored in the membrane of the Golgi apparatus and expressed differentially in cells and tissues such as human placenta, kidney and liver [8,10,19,20]. The soluble form of GalNAcT2 has been successfully produced in both Sf9 insect cells using a baculovirus vector as well as in yeast strains and the activity of the purified transferase has been confirmed [8,18,20,21]. More recently, it has been shown that insect-cell derived GalNAcT2 can be used for *in vitro* glycosylation of the clinically important drug granulocyte colony stimulation factor (G-CSF) [22].

In this work, we describe the expression of functional human-derived glycosyltransferase HisDapGalNAcT2 [8] together with redox folding helpers [16] in the cytoplasm of a recombinant *E. coli* strain. Our findings are a first step towards establishing a glycosylation system in *E. coli* for the transfer of GalNAc to G-CSF.

## Results

### Expression of HisDapGalNAcT2 in *E. coli* host strains

**Figure 1:**
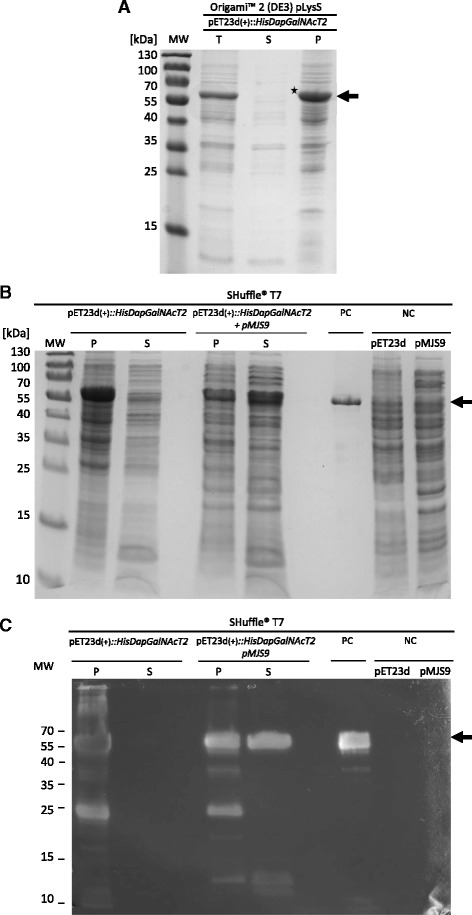
**SDS-PAGE and immunoblot analysis of HisDapGalNAcT2 expressed in** ***E. coli*** **. (A)** Origami™ 2(DE3)pLysS cells carrying plasmid pET23d(+)::*HisDapGalNAcT2* were grown in LB medium at 37°C until OD_600_ 0.5, at which point IPTG (final concentration 1 mM) was added and cultures were incubated for a further 5 h. Cells were harvested by centrifugation, lysed and total cell lysate (T), the soluble protein fraction (S) and the insoluble particulate fraction (P) were separated by SDS-PAGE and visualised by Coomassie staining. HisDapGalNAcT2 with an estimated mass of 61.7 kDa (indicated by arrow) was not detected in the soluble (S) cell fraction, but a band of the correct size in the insoluble particulate (P) fraction (★) was excised and further analysed by ESI-MS. **(B)** SHuffle® T7 cells harbouring either pET23d(+)::*HisDapGalNAcT2* in the absence or presence of pMJS9 were grown in EnPresso B medium. Fractionated cell samples were separated by SDS-PAGE and visualised by Coomassie staining **(B)** or immunoblotting **(C)** using a mouse anti human GALNT2 antibody. Molecular weight markers (MW) are in kDa. HisDapGalNAcT2 with an estimated mass of 61.7 kDa (arrow) was detected in soluble (S) and particulate (P) cell fractions. Commercially available rhGalNAcT2 (PC) and cell lysates of SHuffle® T7 pET23d(+) and SHuffle® T7 pMJS9 cells (NC) were included as controls.

GalNAcT2 is a cysteine rich protein, with five disulfide bonds maintaining the tertiary structure of both the mature [PDB:2FFV] and truncated soluble forms [8,10]. Initial experiments to express the N-terminal fusion of the soluble form of GalNAcT2 lacking the N-terminal transmembrane domain [8] were performed using the *E. coli* expression strain Origami™ 2(DE3)pLysS, which carries mutations in both the thioredoxin reductase (*trxB)* and glutathione reductase (*gor*) genes to provide a less reducing cytoplasmic environment and promote formation of disulfide bonds. Whole cell lysates, soluble and particulate fractions were analysed by SDS-PAGE and a prominent protein band with an apparent molecular weight of 60–64 kDa was identified in whole cell lysates and pelleted material (Figure 1A). Soluble protein was not detected. The protein band of interest was excised and identified as HisDapGalNAcT2 using ESI MS analysis (data not shown).

To enable expression of soluble HisDapGalNAcT2, the *E. coli* host strain SHuffle® T7 was used [3]. In addition to the mutations in *trxB* and *gor,* this expression strain carries a chromosomally integrated gene encoding the disulfide bond isomerase DsbC [3]. Expression experiments with *E. coli* SHuffle® T7 carrying pET23d(+)::HisDapGalNAcT2 were performed. In the SDS-PAGE assay the expected protein band was visible in pelleted cell fractions but soluble protein was not detected (Figure 1B). The result was confirmed by immunoblot analysis (Figure 1C). To further promote the production of soluble HisDapGalNAcT2, the codon usage optimized genes for redox folding helper proteins sulfhydryl oxidase Erv1p and protein disulfide isomerase PDI (Lloyd W. Ruddock, pers. communication) encoded by the pLysS [16] derived construct pMJS9 were pre-expressed under the control of an arabinose promoter [16] and the pET23d(+)::HisDapGalNAcT2-derived glycosyltransferase was subsequently induced. The presence of soluble HisDapGalNAcT2 was detected in the soluble cell fraction by SDS-PAGE and immunoblot analysis (Figure 1B and C).

### Protein purification

**Figure 2:**
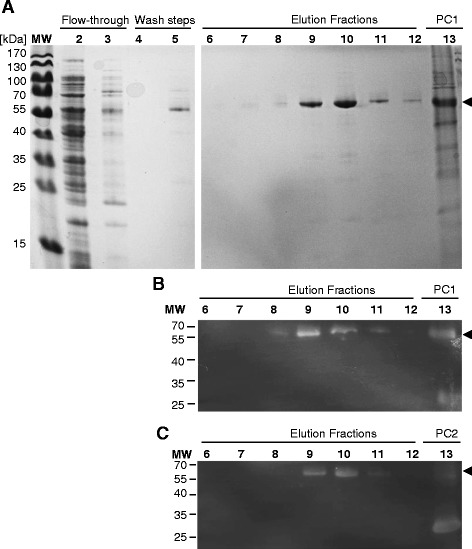
**Purification of HisDapGalNAcT2.** SDS-PAGE **(A)** and immunoblot analyses **(B and C)** of flow-through, wash and elution fractions collected during Ni^2+^-NTA purification (see Materials and methods) of soluble HisDapGalNAcT2. Insoluble HisDapGalNAcT2 (PC1) produced in Origami™ 2(DE3)pLysS and verified by ESI-MS or His-Em-GFP (PC2, 27 kDa) are included as controls. **(A)** Coomassie staining detected protein bands with an expected molecular weight (MW) of *ca.* 61.7 kDa in lanes 7–12. HisDapGalNAcT2 (arrows) was identified by immunoblotting using **(B)** specific polyclonal anti human GALNT2 antibody or **(C)** penta-His antibody against the N-terminal His-Tag.

Several parameters were tested to establish optimal conditions for purification of soluble HisDapGalNAcT2. Preliminary experiments indicated that binding of HisDapGalNAcT2 to Ni-NTA spin columns was impaired by small amounts of imidazole. Omission of imidazole from early steps of the purification protocol resulted in improved binding of the soluble protein to the resin. Immobilised metal affinity chromatography (IMAC) using cobalt or nickel resins proved feasible (data not shown), and in subsequent experiments Ni-NTA resin was used, as described in the methods section. Fractions eluted from the resin were collected, proteins were separated by SDS-PAGE (Figure 2) and bands of approx. 60 kDa visible by Coomassie staining were confirmed to be glycosyltransferase by immunoblotting (Figure 2B, lanes 8–12 and Figure 2C lanes 9 to 11). Eluted fractions 9, 10 and 11 were pooled and determined to have a protein concentration of 0.86 mg/ml by BCA protein assay. This pooled sample with an estimated yield of 0.32 mg HisDapGalNAcT2 per gram of cell pellet was used for further analysis.

### Analysis of purified HisDapGalNAcT2 by size exclusion chromatography (SEC) and multi angle light scattering (MALS)

**Figure 3:**
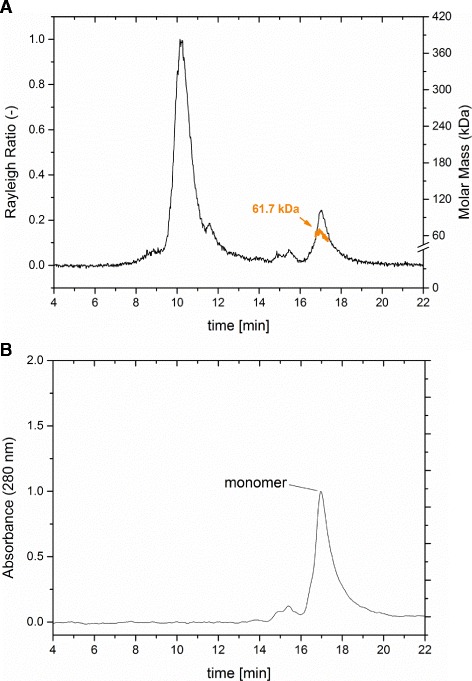
**SEC-MALS analysis of the purified HisDapGalNAcT2 protein. (A)** Light scattering spectrum of the eluted glycosyltransferase fraction in 50 mM Tris, 150 mM NaCl, pH 8. The orange line across the peak at the retention time of 17 min shows the molar mass distribution. **(B)** The UV-spectrum measured at 280 nm shows a single peak representing monomers of HisDapGalNAcT2.

Size exclusion chromatography with an inline multi-angle light scattering detector (SEC-MALS) was used to assess the presence of monomers, dimers and multimers in the purified protein sample. The light scattering spectra revealed a peak with 10.5 min retention time followed by a second peak after 17 min (Figure 3A). The SEC-MALS method was used to determine the molar mass across the peak at 17 min retention time and the result of 61.7 (±1.2) kDa was as expected for soluble monomeric HisDapGalNAcT2 (Figure 3A). Pairing this method with ultraviolet spectroscopy (UV), the protein content in the eluted fraction was recorded at 280 nm wavelength. The results of the UV spectrum indicate a low content of proteins with a molecular mass other than 61.7 (±1.2) kDa in the purified sample (Figure 3B).

### Circular dichroism (CD) spectroscopy analysis

**Figure 4:**
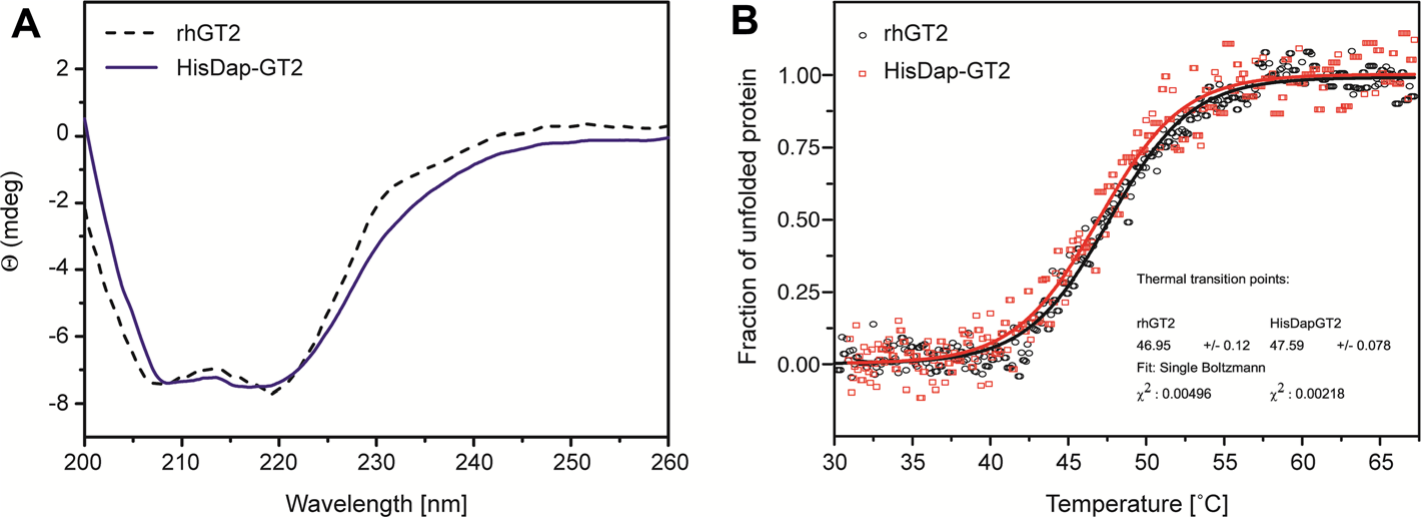
**CD spectroscopy and thermal stability measurements of HisDapGalNAcT2 in comparison to rhGalNAcT2. (A)** Far UV (195–260 nm) spectra of HisDapGalNAcT2 (solid line) and rhGalNAcT2 (dashed line) indicating the folded status of the proteins at 21°C in 20 mM sodium phosphate buffer at pH 7.3 CD data are reported as molar ellipticity (Θ) [24]. **(B)** Thermal unfolding profiles of HisDapGalNAcT2 (squares) and rhGalNAcT2 (circles) were monitored at 208 nm.

The secondary structure and thermal transitions of HisDapGalNAcT2 were assessed by CD spectroscopy [23,24]. CD scans at far UV wavelengths (195-260 nm) identified two characteristic minima at 208 nm and 220 nm corresponding to α-helical protein features (Figure 4), indicating that the purified HisDapGalNAcT2 was folded. This notion was supported by the finding that commercially available rhGalNAcT2 from NS0 murine myeloma cells produced similar CD spectra (Figure 4A). Thermal unfolding profiles of both glycosyltransferases were measured by recording temperature-dependent molar ellipticity changes [24] at 220 nm between 30°C and 65°C after heating the respective protein solution. Based on the sigmoid graphs shown in Figure 4, the thermal transition points of HisDapGalNAcT2 and rhGalNAcT2 were determined at 47.59°C (±0.07) and 46.95°C (±0.12), respectively. These data indicate a difference of about one degree with a higher transition point for HisDapGalNAcT2 compared to the commercially available rhGalNAcT2 (Figure 4B), suggesting similar characteristics of the HisDapGalNAcT2 purified from *E. coli* and the glycosyltransferase produced in NS0 cells.

### Analysis of HisDapGalNAcT2 enzymatic activity

**Figure 5:**
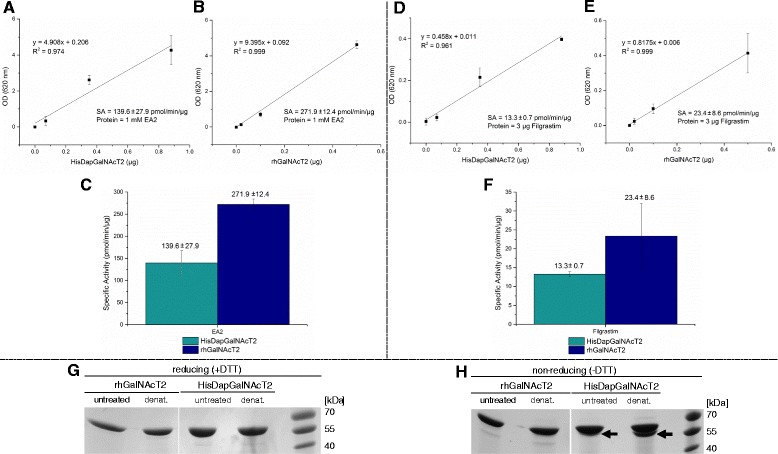
**SDS-PAGE analysis and specific activity (SA) of HisDapGalNAcT2 compared to rhGalNAcT2 using the acceptor peptide EA2 or Filgrastim™.** All reactions were initiated with 0.2 mM UDP-GalNAc and 1 mM EA2 or 3 μg Filgrastim™ per well and proceeded at 37°C for 60 min. **(A, B, D, E)** Absorbance at 620 nm plotted against glycosyltransferase input in μg. **(C, F)** Comparison of the SA of HisDapGalNAcT2 and rhGalNAcT2. Denatured (5 min at 95°C) and untreated (10 min at room temperature) protein samples of both glycosyltransferases were separated by either reducing **(G)** or non-reducing **(H)** SDS-PAGE. The emerging double band is indicated by an arrow.

**Figure 6:**
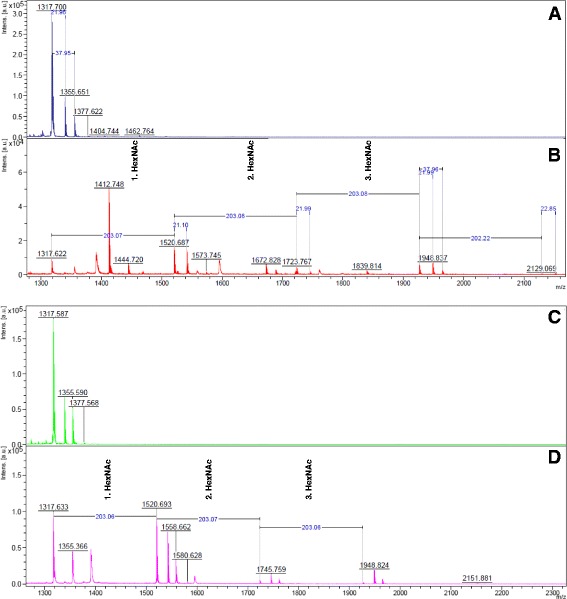
**MALDI-TOF-MS analysis of the glycosylation pattern of mucin EA2.** MS spectra of **(A, C)** non-glycosylated EA2 in the absence of glycosyltransferases and EA2 after glycosylation by either **(B)** HisDapGalNAcT2 or **(D)** rhGalNAcT2. N-acetylgalactosamine residue (HexNAc).

To assess whether HisDapGalNAcT2 was enzymatically active, a colorimetric glycosyltransferase assay was used [25] (Figure 5). In this assay, N-acetylgalactosamine derived from the sugar substrate UDP-GalNAc is added to an acceptor substrate with the concomitant release of nucleotide diphosphate UDP. A specific coupling phosphatase then hydrolyses UDP and the quantitative release of phosphate is detected using malachite based reagents. Using peptide EA2 (PTTDSTTPAPTTK [10]) as a substrate, the specific activities of HisDapGalNAcT2 and rhGalNAcT2 were measured as pmol of phosphate released over time per μg of glycosyltransferase (pmol/min/μg; Figure 5A-B). HisDapGalNAcT2 was found to be an active glycosyltransferase with a specific activity of 139.6 ± 27.9 pmol/min/μg, approximately half the specific activity measured for the commercially available rhGalNAcT2 (271.9 ± 12.4 pmol/min/μg) (Figure 5C). To confirm that the acceptor substrate had indeed been glycosylated by the glycosyltransferases, MALDI-TOF-MS was performed (Figure 6A-D). Analysis of the un-glycosylated EA2 acceptor peptide generated peaks with m/z of 1317.7, consistent with its predicted molecular weight of 1317.4 Da [26,27]. Peaks shifted by m/z 22 and 38 are consistent with sodium and potassium adducts of the substrate, respectively (Figure 6A). Analysis of EA2 peptides from glycosyltransferase assays containing HisDapGalNAcT2 (Figure 6B) or rhGalNAcT2 (Figure 6D) revealed new peaks with m/z of 1520.7, 1723.8 and 1926.9, consistent with the addition of the monosaccharide N-acetylgalactosamine (HexNAc; m/z 203) at one, two or three sites in the peptide substrate.

**Figure 7:**
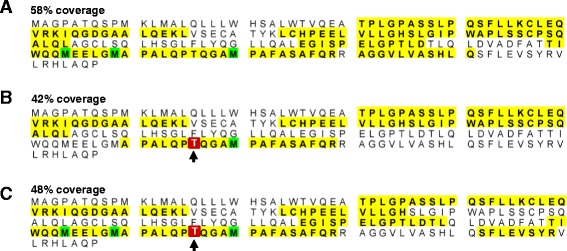
**ESI-MS/MS analysis of non-glycosylated and glycosylated Filgrastim™ (G-CSF). (A)** Amino acid sequence of the substrate Filgrastim™ showing the tryptic digest peptides detected by ESI-MS/MS (yellow highlight, 58% coverage) and post-translationally modified amino acids (e.g. oxidation, green). G-CSF peptides detected by ESI-MS/MS after treatment with glycosyltransferases **(B)** HisDapGalNAcT2 (42% coverage) or **(C)** of rhGalNAcT2 (48% coverage), showing the glycosylation site at Thr^166^ (red box and black arrow).

The glycosyltransferase assay was then extended to test glycosylation of a biopharmaceutically relevant substrate, the granulocyte-colony stimulation factor (G-CSF) Filgrastim™ [UniProt:P09909], which contains only one putative O-glycosylation site (Figure 5D-E). The specific activity of HisDapGalNAcT2 was found to be 13.3 ± 0.7 pmol/min/μg, again approximately half the specific activity measured for rhGalNAcT2 (23.4 ± 8.6 pmol/min/μg; Figure 5F). The presence of glycosylated Filgrastim™ in assay samples was confirmed using ESI-MS analysis (Figure 7). These results indicate that both glycosyltransferases, HisDapGalNAcT2 and rhGalNAcT2, transfer N-acetylgalactosamine to the hydroxyl group of Thr^166^ in Filgrastim™ (Figure 7).

To assess partial misfolding of the protein e.g. due to incorrectly linked disulfide bonds as a factor contributing to the difference in the specific activities measured, samples of purified HisDapGalNAcT2 and commercially available rhGalNAcT2 were investigated employing SDS-PAGE assays under reducing and non-reducing conditions (Figure 5G-H). Denatured as well as untreated HisDapGalNAcT2 and rhGalNAcT2 migrate as homogeneous single band at about 61.7 kDa in reducing SDS-Gels (Figure 5G). Differences in the migrating behaviour of the proteins were observed with a double band emerging under non-reducing conditions, an effect even more pronounced in the denatured sample of HisDapGalNAcT2. Using densitometric analyses the lower band represents an estimated 42.1% and the upper band 57.9% of the total protein content under non-reducing, denaturing conditions. Only a weak double band was visible for rhGalNAcT2 (Figure 5H). This result indicates the presence of different protein configurations in the protein samples, however, the activity of purified HisDapGalNAcT2 was clearly demonstrated.

## Discussion

The expression of complex, correctly folded recombinant proteins stabilised by disulfide bonds within the cytoplasm of *E. coli* remains a challenging approach [3]. To isolate a functional human-derived glycosyltransferase the *E. coli* host strain Origami™ 2(DE3)pLysS proved unsuccessful. Subsequent experiments were carried out using the expression strain SHuffle®T7, a derivative of the *E. coli trxB gor* suppressor strain SMG96, which carries mutations both in the thioredoxin reductase (*trxB*) and gluthathione reductase (*gor*) [28]. The correct folding of the expressed protein in an *E. coli* SHuffle® T7 strain background is therefore aided by a less reducing cytoplasm and the presence of a chromosomal copy of the disulfide bond isomerase DsbC [3]. The strain has been engineered to cytoplasmically express DsbC lacking its signal sequence that targets the protein to the periplasm [3]. The correct folding and soluble expression of recombinant proteins has been further improved by including helper proteins [3,16]. As an example, work published by Skretas *et al*. described the co-expression of chaperones leading to improved production of recombinant disulfide-bonded human sialyltransferase ST6GalNAcI in *E. coli* [1]. However, co-expression of trigger factor, DnaK/DnaJ, GroEL/GroES, and Skp did not always result in increased soluble yields of ST6GalNAcI in engineered bacterial strains [1]. The expression strain *E. coli* SHuffle® T7 pET23d(+)::*HisDapGalNAcT2* failed to show an improvement and only insoluble HisDapGalNAcT2 was identified in pelleted cell material. To produce soluble HisDapGalNAcT2 in *E. coli* the expression system was further optimised. The pre-expression of PDI and Erv1p was required for the production of soluble HisDapGalNAcT2 in the *E. coli* strain indicating that the presence of DsbC in the cytoplasm, as demonstrated by the SHuffle® T7 strain without pMJS9, is not sufficient. During the course of the expression experiments described in this paper, the combination of SHuffle® T7 as expression strain, the pre-expression of Erv1p and PDI [16], and the use of EnPresso B medium for controlled glucose release [29-31] proved to be successful. The calculated molar mass of 61.7 kDa and the monomer status of the purified HisDapGalNAcT2 were confirmed by SEC-MALS analysis. It has been suggested that the soluble forms of glycosyltransferases such as GalNAcT2 appear to be in the monomer form [8]. CD-spectroscopy measurements were performed comparing isolated HisDapGalNAcT2 with the commercially available rhGalNAcT2 and the results indicate a similar folding state for both glycosyltransferases, a finding that is further supported by the observed similar thermal transition points. The presence of different protein configurations in the protein samples were observed in SDS-PAGE-assays carried out under non-reducing conditions.

This result might help to explain the specific activity of the tagged HisDapGalNAcT2 determined in the glycosyltransferase assay which was lower than that of rhGalNAcT2. Other potential contributing factors include differences in the N-terminal his-tag fusions of the glycosyltransferases and storing conditions employed. The N-terminal sequence of the glycosyltransferases is not identical with a hexa-His-Tag translationally linked to rhGalNAcT2 and a larger slightly alkaline N-terminal HisDap purification tag comprising 17 amino acids present in HisDapGalNAcT2, which might result in the lower specific activity observed for this protein. Although there is one potential glycosylation site in GalNAcT2, glycosylation does not seem to be critical for function as the unglycosylated form is enzymatically active [8,21,32]. Therefore, lack of glycosylation might not be the cause of the lower activity observed for GalNAcT2 purified from *E. coli*.

The mucin type O-glycosylation of the acceptor substrates EA2 and Filgrastim™ were analysed by mass spectroscopy. The results indicate the presence of one to three monosaccharide residues consistent with N-acetylgalactosamine on the peptide EA2 and the glycosylation of Thr^166^ of Filgrastim™. The association between human GalNAcT2 and peptide substrate EA2 derived from rat submandibular mucin [UniProt:Q62605] has been studied in detail and the catalytic mechanism involved has been used as a model of O-glycosylation by retaining glycosyltransferases [27,33]. GalNAcT2 has been grouped among the early transferases that prefer non- and monoglycosylated peptide substrates and are consequently responsible for beginning stages of mucin glycosylations [27]. Using a glycopeptide library it has been shown that GalNAcT2 accepts various EA2 derived peptides carrying different glycosylation patterns as substrates [27]. The preferred site for initial glycosylation has been identified as the side chain hydroxyl of threonine at position 7 of the EA2 peptide substrate [10,27].

Six different human recombinant GalNAc-transferases have been studied identifying GalNAcT2 as the only candidate for glycosylation of G-CSF [22]. The O-glycosylation at Thr^166^ is not required for potency [34]. Filgrastim™, the non-glycosylated form of recombinant G-CSF, is produced in *E. coli* and used to reduce the duration of neutropenia in patients treated with cytotoxic chemotherapy and in patients undergoing therapy followed by bone marrow transplantation [35]. It has been concluded that the O-linked sugar chain decreases polymerisation and contributes to the stability of G-CSF [36]. Experiments are in progress to compare the expression of G-CSF in the presence and absence of HisDapGalNAcT2 studying the potential impact of a glycosyl residue at Thr^166^ in *E. coli*.

Based on previously published results it has been suggested that the glycosyltransferase displays a somewhat relaxed specificity towards an alternative sugar substrate [21,33]. The authors concluded that UDP-Galactose, also available in *E. coli*, might serve as a naturally relevant substrate for GalNAcT2 [6,21,33]. Based on the results reported in this paper, *in vivo* experiments are under way to study the transfer of glycosyl residues to the acceptor substrates EA2 and Filgrastim™ in *E. coli*.

## Conclusion

The results presented here provide clear evidence for the expression of an N-terminal His-tagged functional human-derived glycosyltransferase UDP-GalNAc:polypeptide N-acetylgalactosaminyl-transferase 2 (HisDapGalNAcT2) in *E. coli*. The expression system described in this work combines the use of *E. coli* strain SHuffle® T7 with a less reducing cytoplasm and the co-expression of redox folding helpers leading to the production of soluble HisDapGalNAcT2 in special production medium. The glycosyltransferase was isolated, the folding status of the HisDapGalNAcT2 was assessed and the activity of the protein in *in vitro* assays was shown. Further investigations to study the *in vivo* activity of HisDapGalNAcT2 in *E. coli* are ongoing. The experiments in this paper represent an approach to improve the understanding of *E. coli*-based expression systems for the production of human glycoproteins and to widen the future use of engineered *E. coli* strains as cell factories for the production of recombinant glycoproteins.

## Materials and methods

### Bacterial strains

The *Escherichia coli* strains NovaBlue (Novagen, Merck Millipore, Germany), SHuffle®T7 (C3026H, New England Biolabs, Germany) and Origami™ 2(DE3)pLysS (Novagen, Merck Millipore, Germany) were routinely grown in LB medium and maintained under standard conditions [37]. *E. coli* transformants were selected with 120 μg/mL ampicillin and 34 μg/mL chloramphenicol as indicated. Protein expression was carried out using EnPresso B medium [29-31] including booster tablets following protocols provided by the manufacturer (BioSilta Oy, Finland). Unless otherwise stated, chemicals and reagents were obtained from Sigma-Aldrich or Roth (Carl Roth GmbH & Co. KG, Germany).

### Plasmids

Plasmid pET-23d was obtained from Merck Millipore (Germany) and plasmid pET23d(+)::*HisDap* was constructed encoding a diaminopeptidase-cleavable histidine-tag [37]. DNA was prepared using the Roti®-Prep Plasmid MINI kit (Carl Roth GmbH & Co. KG, Germany) and DNA fragments were isolated from agarose gel blocks employing the QIAquick Gel Extraction Kit (Qiagen, Germany). Plasmid pMJS9 [16] was kindly provided by Prof. Dr. L. W. Ruddock.

A gene sequence was designed to express amino acids 52–571 [8] of the human N-acetylgalactosaminyltransferase 2 [GalNT2, GeneID:2590, UniProtID:Q10471] and the restriction sites *Nde*I and *Xho*I were added at the 5′ and 3′ end of the DNA fragment, respectively. As a result, an additional methionine residue followed by an alanine moiety was added to the N-terminal sequence. The codon usage of the gene fragment was adapted for expression in an *Escherichia coli* strain background [38]. The synthesised DNA fragment was purchased from GeneArt® Gene Synthesis and supplied as plasmid construct cloned into the pMK-RQ shuffle vector (Life Technologies GmbH, Germany). Plasmid-DNA was digested with *Nde*I and *Xho*I, the 1.8 kb fragment was isolated and ligated with vector pET23d(+)::*HisDap* treated with the same restriction enzymes. The DNA was subsequently used to transform *E. coli* NovaBlue. As a result, the decahistidine-tag with the sequence MAHHHHHQHQHQHQHQH was translationally fused to the N-terminus of the expressed protein thus facilitating purification of the product. The resulting construct pET23d(+)::*HisDapGalNAcT2* was verified by restriction digests and the cloned fragment was confirmed by DNA sequence analysis.

### Protein expression of HisDapGalNAcT2

Plasmid pET23d(+)::*HisDapGalNAcT2* was first expressed in Origami™ 2(DE3)pLysS and the production of insoluble HisDapGalNAcT2 was detected. The bacterial suspension was lysed by using ultrasonic sound. Afterwards the whole cell lysate was centrifuged to separate the soluble fraction (supernatant) from the particular fraction (cell pellet). Whole cell lysate, supernatant and particulate fraction were analysed by SDS-PAGE. Subsequent experiments were carried out with *E. coli* SHuffle® T7 as strain background.

Plasmids pET23d(+)::*HisDapGalNAcT2* and pMJS9 were used to transform and co-transform the *E. coli* host strain SHuffle® T7. Plasmid pMJS9 encodes the sulfhydryl oxidase Erv1p and the protein disulfide isomerase PDI under the control of the arabinose-promoter [16]. Transformants were grown on LB agar plates supplemented with the required antibiotics and a single colony was used to inoculate a 2 mL liquid culture. Following incubation, culture aliquots were frozen at −80°C employing the CRYOBANK™ system (CRYOBANK™, Mast Diagnostica GmbH, Germany). For expression experiments two ceramic beads were transferred from a cryovial into a 50 mL Falcon tube containing 2 mL LB media (120 μg/mL ampicillin, 34 μg/mL chloramphenicol and 0.2% glucose) and the cells were maintained at 37°C, 175 rpm for 8 h. The culture was transferred into a 2 L baffled flask containing 200 mL EnPresso B medium (120 μg/mL ampicillin, 34 μg/mL chloramphenicol, 100 μL Reagent A) and grown at 30°C, 175 rpm for 15 h. Booster solution was added following the manufacturer’s instructions. The pre-induction of the pMJS9 encoded gene products was carried out in the presence of 0.5% w/v arabinose. Isopropyl-β-D-thiogalactopyranosid (IPTG) was added after 30 min to a final concentration of 1 mM to induce expression of the glycosyltransferase HisDapGalNAcT2 and the culture was incubated at 30°C, 175 rpm for 23.5 h. The cells were harvested and 10 mL fractions were pelleted via centrifugation at 4°C, 5500 g for 5 min. The supernatants were discarded and the cell sediments stored at −80°C. A scaled-up version of this experiment was carried out and a 15 mL culture was prepared to inoculate 1.5 L expression medium in a 2 L bioreactor (BIOSTAT®Bplus, Sartorius Stedim Biotech, Germany) at 30°C, 60% pO_2_, 0.5 vvm, pH 7, adopting conditions and procedures already described. Cell pellets were pooled and stored at −80°C. A 7.98 g portion of the cell sediment was used for protein purification.

### Protein purification

A cell pellet derived from a 10 mL fraction of the expression culture was resuspended in 1.26 mL extraction buffer (50 mM Tris, 300 mM NaCl; pH 8) containing 140 μL lysozyme, 3 μL DNAse and 50 μL protease inhibitor (complete protease inhibitor cocktail tablet, F. Hoffmann La-Roche AG, Switzerland). The bacterial suspension was cooled on ice for 30 min and sonicated for 3 min on ice. The cell lysate was centrifuged at 4°C, 16 100 g for 10 min and the supernatant was passed through a 0.45 μM filter. The polyhistidine-tagged protein HisDapGalNAcT2 was purified using Ni-NTA spin columns (Qiagen, Germany) with washing buffer (50 mM Tris, 300 mM NaCl, 20 mM imidazole) and elution buffer (50 mM Tris, 300 mM NaCl, 500 mM imidazole) adjusted to pH 8 prior to use.

Similarly, 29 mL lysate were generated using a 7.98 g portion of the previously isolated cell pellet. HisDapGalNAcT2 was purified on a HisTrap HP 1 mL column with an ÄKTA Purifier (GE Healthcare Life Sciences). The column was equilibrated with 8 column volumes (CV) equilibration buffer (50 mM Tris, 300 mM NaCl; pH 8) and the 29 mL sample was loaded, followed by 8 CV of washing buffer (50 mM Tris, 300 mM NaCl, 20 mM imidazole; pH 8). The protein was eluted (50 mM Tris, 300 mM NaCl, 500 mM imidazole; pH 8) using a linear gradient from 20 mM to 500 mM imidazole over 4 CV. 1 mL fractions were collected for further analysis.

### Analysis of purified protein

SDS-PAGE and immunoblot analyses were carried out using standard protocols with dithiothreitol (DTT) as reducing agent. Densitometric analysis was performed employing Fusion-FX software (Vilber Lourmat, Germany). For Western blot analysis, proteins were transferred to polyvinylidene fluoride membranes (PVDF, Thermo Scientific Inc., USA) and treated with mouse anti human GALNT2 antibodies (H00002590-A01, Acris Antibody GmbH, Germany) or penta-His antibodies (34660, Qiagen, Germany). Binding was detected with horseradish peroxidase (HRP)-labeled secondary antibodies and chemiluminescence was assessed using Roti®Lumin solutions.

Purified proteins were analysed by size exclusion chromatography coupled to multi-angle light scattering (SEC-MALS) and circular dichroism (CD) spectroscopy to determine molecular mass, size, aggregation state/dispersity, folding state and thermal transitions of the protein [23,24]. Proteins were separated according to different molecular masses using a size exclusion column (SEC) (Yarra SEC-2000, Phenomenex Inc., Germany) on a Multi-Angle static Light Scattering (MALS) detector (Dawn®8+ HELEOS, 663.9 nm) in combination with an ÄKTA Explorer (GE Healthcare Life Sciences) and protein concentrations of the fractions were measured (Optilab® T-rEX Refractive index detector, 658 nm, 25°C, and ASTRA® Software, Wyatt Technology Europe GmbH, Germany). 100 μL of the purified and filtrated samples (0.2 μm, polyethersulfone membrane) were injected at a concentration of 0.13 mg/mL in running buffer (50 mM Tris, 300 mM NaCl, pH 8.0). Flow rate was adjusted to 0.5 mL/min.

Spectra of a filtered solution (NanoSep® MF 0.2 mM, Pall) containing 0.2 mg/mL HisDapGalNAcT2 or rhGALNT2 in 20 mM sodium phosphate buffer (pH 7.3) were recorded in a 715 CD-spectropolarimeter (Jasco, Hachioji, Japan) at 25°C. Spectra were measured from 190 – 260 nm with wavelength steps of 0.1 nm and a scan speed of 50 nm per minute. The averaged signal from four scans was corrected for the buffer signal. Thermal transitions were recorded at 208 nm with a step size of 0.1°C and a thermal slope of 1°C per minute. The data was fitted using a Boltzmann equation $y=\frac{{\mathrm{A}}_1\hbox{-} {\mathrm{A}}_2}{1+e\left(x-{x}_0\right)/dx}+{\mathrm{A}}_2$ to obtain the transition point (OriginPro 8, OriginLab, Northampton, Massachusetts, USA).

### Protein activity assay

The activity of HisDapGalNAcT2 was monitored using a glycosyltransferase activity kit (EA001, R&D Systems Europe Ltd., United Kingdom) based on the release of phosphate during transfer of glycosyl residues to an acceptor peptide. UDP-N-acetylgalactosamine was used as donor substrate in combination with the acceptor substrates mucin EA2 (Eurogentec SA Anaspec, Belgium) comprising the peptide sequence PTTDSTTPAPTTK [10] or Filgrastim (Neupogen®, Amgen Inc.). Prior to use, the elution buffer of the purified HisDapGalNAcT2 (50 mM Tris, 300 mM NaCl, 500 mM imidazole; pH 8) was exchanged by 25 mM Tris, 150 mM NaCl, pH 7.3. The protein sample was stored at 4–8°C for about 3 weeks. Control assays were carried out using purified rhGALNT2 isolated from murine myeloma NS0 cells (7507-GT-020, R&D Systems Europe Ltd., United Kingdom). The enzyme was provided by the manufacturer in 25 mM Tris, 150 mM NaCl, 0.05% w/v Brij-35; pH 7.5 and stored in the freezer at −80°C.

Samples with EA2 as acceptor substrate were desalted, concentrated (ZipTip®_μC18_, Millipore, USA) and embedded into a HCCA matrix (α-cyano-4-hydrosycinnamic acid) for subsequent MALDI-TOF-MS analysis. Filgrastim samples were digested with trypsin prior to Electrospray Ionisation Mass Spectrometry (ESI-MS). Both analyses were carried out at the life science center of the University of Hohenheim (Germany).
